# Supplementary material for: Examining person-centered maternity care in a peri-urban setting in Embakasi, Nairobi, Kenya
Source: PLoS One. 2021 Oct 11;16(10):e0257542. doi: 10.1371/journal.pone.0257542 (PMC8504752; doi:10.1371/journal.pone.0257542)
Supplement: S1 Appendix — (DOCX) [file pone.0257542.s001.docx]

**PARTICIPATING HOSPITALS**

|  | FACILITY | FACILITY TYPE | NUMBER OF WOMEN INTERVIEWED |
| --- | --- | --- | --- |
| 1. | Mama Lucy Kibaki Hospital | Public | 118 |
| 2. | Ruben Centre Clinic | Faith based | 113 |
| 3. | Pipeline Nursing Home | Private | 33 |
| 4. | Samaritan Medical Services | Private | 20 |
| 5. | Mkunga Hospital | Private | 7 |
| 6. | Dandora Medical Centre | Private | 6 |
| 7. | Provide International Clinic | Private | 5 |
| 8. | Paradise Health Clinic | Private | 5 |
|  | **Total** |  | **307** |
