# Supplementary material for: Examining person-centered maternity care in a peri-urban setting in Embakasi, Nairobi, Kenya
Source: PLoS One. 2021 Oct 11;16(10):e0257542. doi: 10.1371/journal.pone.0257542 (PMC8504752; doi:10.1371/journal.pone.0257542)
Supplement: Attachment — Submitted filename: Review- pone-d-21-00183.docx [file pone.0257542.s003.docx]

Md. Arif Billah

Department of Psychology and Counselling

Faculty of Business, Economic and Social Development

Universiti Malaysia Terengganu

To the editor

The manuscript is well formatted and represent the person-centered maternity healthcare in peri-urban area of Kenya. The concept is quite interesting and up to date with the recent health care services. The authors describe the use of pre-developed scales, sampling and result discussion which is very effective and ease to understand. Overall, the work is great except some following problems-

1. Peri-urban area is mainly worked as the bridge of rural and urban area. So, basically there must be some difference in the maternal health-facilities. In the introduction part, if the author(s) can add a brief rationale of why he(they) uses the peri-urban area for person centered maternity healthcare, the study might be justified by the study location.
2. Several part of the manuscript can be modified to represent the concise information, specially, in the introduction and statistical analysis of methodology part. Note that, several repetitive words are seen in the manuscript, in the consequences, several parts are poorly matched with the total manuscripts (for example, please see the 3^rd^ and 4^th^ paragraph of the introduction section and full methods section).
3. The author can describe the person-centered maternity care. Although he defined it with the dimensions that constructed from two papers, but a brief explanation of such dimensions could be included.
4. In table 1, the number of never married is 45 while in education level of partner implies that there are 41 counts have no partner. In this case, the author should explain the bit about the variation. Again, what is pregnancy complication and severe pregnancy complications? The queries of how the degree of pregnancy complication become severed when already pregnancy complication reported? The author should delete one of the variables or clarify the use of similar variables. Lastly, in the variable, type of facility, the author(s) mentions the mission is one of the types while in the methods part he mentions the faith-based facilities. In this regard, the author(s) should be more consistent with the variables and the labels.
5. Table 2 can be regenerated by increasing the columns instead of rows. Additionally, table 3 can be integrated with the table 2.
6. The conclusion should be more result oriented.
7. Grammatical and repetitive words should be declined to the concise information related to the study objectives and results.
