# Supplementary material for: Examining person-centered maternity care in a peri-urban setting in Embakasi, Nairobi, Kenya
Source: PLoS One. 2021 Oct 11;16(10):e0257542. doi: 10.1371/journal.pone.0257542 (PMC8504752; doi:10.1371/journal.pone.0257542)
Supplement: Attachment — Submitted filename: PCMC Quantitative Paper- Responses to Reviewers_Final.docx [file pone.0257542.s004.docx]

**RESPONSES TO REVIEWER #1**

|  | **COMMENTS** | **RESPONSES** | **Section in the Manuscript** |
| --- | --- | --- | --- |
| 1. | Peri-urban area is mainly worked as the bridge of rural and urban area. So, basically there must be some difference in the maternal health-facilities. In the introduction part, if We can add a brief rationale of why he (they) uses the peri-urban area for person centered maternity healthcare, the study might be justified by the study location. | We concur with the reviewer that there are some differences in terms of maternal health facilities serving peri-urban settings and urban/rural areas. We have included a brief rationale in the introduction to clarify on why we focused our study within a peri-urban setting. This is mainly related to differences in quality of maternal health services. See pasted below:  *“...Although previous studies have examined PCMC in both rural and urban settings in Kenya, little work has been done in peri-urban settings. Peri-urban settings in Kenya are often close to cities, but lack access to basic amenities such as running water and adequate sanitation. The healthcare system within these settings has also been reported to be deficient and health facilities are known to provide varied quality of care (17)...”* | Introduction section pg. 3 |
| 2. | Several part of the manuscript can be modified to represent the concise information, specially, in the introduction and statistical analysis of methodology part.  Note that, several repetitive words are seen in the manuscript, in the consequences, several parts are poorly matched with the total manuscripts (for example, please see the 3rd and 4th paragraph of the introduction section and full methods section). | The manuscript has been revised to represent concise information. The introduction and the methodology have been revised to remove the repetitive words.  The third and fourth paragraph of the introduction section as well as the full methods section have been revised | Introduction section pg. 3-5  Methodology section pg. 5-8 |
| 3. | The author can describe the person-centered maternity care. Although he defined it with the dimensions that constructed from two papers, but a brief explanation of such dimensions could be included. | A brief explanation has been included as follows to further describe person centered maternity care :  Person-centered maternity care (PCMC) represents the experience or interpersonal dimensions of quality of care. It refers to care that is respectful and responsive to the needs of women and their families (5). PCMC emphasizes the patient-provider relationship, and highlights issues such whether women are treated in a dignified and respectful manner, are communicated to effectively and feel involved in decision-making about their care, and feel supported both emotionally and socially during childbirth (5). | Introduction section pg.3 |
| 4. | In table 1, the number of never married is 45 while in education level of partner implies that there are 41 counts have no partner. In this case, the author should explain the bit about the variation. | We have provided a note with a brief description about the variable marital status and the educational level of the participants in the research in table 1.  6 respondents are cohabitating, meaning they are never married but they have a partner. 2 respondents are widowed, so they do not have a partner but they were married. This variation explains the difference of 4 respondents between these two variables. This has been briefly mentioned in a footnote. | Pg 8 Foot note |
|  | Again, what is pregnancy complication and severe pregnancy complications? The author should clarify the pregnancy complication and severe pregnancy complication and how he used these two attributes? Otherwise, the author should delete one of the variables or clarify the use of similar variables. | We have provided an explanation distinguishing between pregnancy complications and severe pregnancy complications. There were 23 pregnancy complications that the respondent could have identified, or they had the option to list a complication which was not included in the options of responses. If they listed any complications, they were asked if the problem was severe. So the severity of the problem was self-determined. This has been added in the description of the variables. | Results section pg. 7 |
|  | Lastly, in the variable, type of facility, We mentioned the mission is one of the types while in the methods part he mentions the faith-based facilities. In this regard, Weshould be more consistent with the variables and the labels. | The comment is well received and for the variable type of facility, We will use faith-based as the consistent term to describe health facilities that are run and operated by religious organizations | Throughout the manuscript |
| 5. | Table 2 can be regenerated by increasing the columns instead of rows. Additionally, table 3 can be integrated with the table 2. | Table 2 has been regenerated by increasing the columns as requested.  Unfortunately for table 2 and 3 cannot be integrated because of space | Table 2 pg 13-15 |
| 6. | The conclusion should be more result oriented. | The conclusion has been revised to speak to the results | Conclusion section pg 20 |
| 7. | Grammatical and repetitive words should be declined to the concise information related to the study objectives and results. | The manuscript has been reviewed and the repetitive words have been removed | Throughout the manuscript |

**RESPONSES TO REVIEWER #2**

|  | **COMMENTS** | **RESPONSES** |  |
| --- | --- | --- | --- |
|  | **TITLE** |  |  |
| 1. | This study is completed in one area of Nairobi County, so how can it represent the whole Nairobi County as well as the Kenya? That means how do you measure the whole County and/or Country by only one area/city?  You may rephrase your title as: ‘Examining Person-Centered Maternity Care in peri-urban settings in Embakasi, Nairobi, Kenya’ | To address this concern while keeping the title concise, we have rephrased the title to: ‘Examining Person-Centered Maternity Care in a peri-urban setting in Nairobi, Kenya. | Title page |
|  | **ABSTRACT** |  |  |
| 2. | In the introduction section you have wrote ‘like’ before the word ‘Kenya’. I recommend you to recheck that word and sentence. I think ‘in’ will replace the ‘like’. | We have replaced the word ‘like’ with ‘in’ as advised. | Abstract |
| 3. | In the methods section you have considered the women aged 18-49 years who had delivered a baby within 4 to 6 weeks as the respondents. But you didn’t provide any rationale behind these. Why did you choose 18-49 years, and within 4 to 6 weeks? | We considered women aged 18-49 years because these are women considered to be of reproductive age.  We considered women who had delivered a baby within 4 to 6 weeks because this is when women go back to hospital for their first clinical visits | Pg 5 |
| 4. | What was your logic behind recruiting the women from public (n=118), private (n=76), and faith based (n=113) health facilities? | This sample size was established recruited was based on available health facilities in the setting and a multi stage purposive sampling design strategy that has been discussed in the methodology section |  |
|  | **INTRODUCTION SECTION** |  |  |
| 5. | Authors wrote pointlessly a lot about background profile of the study. Please make it short, simple and precise according to the title. | The background has been revised to make it short and simple | Introduction section |
| 6. | Add some statistics nationally and internationally, and compare them. | Authors have added a comparison to the maternal mortality rate of low income countries that was estimated as 462 per 100, 000 live births and compared it to Kenya’s national mortality rate of 362 per 100,000 to provide a comparison | First paragraph pg 1 |
| 7. | Need a major revision in the Literature review section. | The literature review section has been revised | Introduction section |
| 8. | Draw a clear research gap. | The research gap has been identified as a lack of information on the extent of PCMC in peri-urban settings that are contributing to maternal health deaths | Introduction section pg 1 last paragraph |
|  | **METHODS SECTION** |  |  |
| 9. | Is this study a longitudinal or cross-sectional study? In abstract section you wrote it is cross-sectional but in methods section you have mentioned it is longitudinal. So this is confusing. Make it clear. | This has been corrected, the study was a cross sectional study | Page 5 |
| 10. | Add the reference(s) after the following sentences:  The area is characterized by low-income ……. access to water and waste disposal.  The health system within Embakasi ……health facilities and faith-based health facilities.  The main referral health facility for …… that is situated in Embakasi-West.  We divided the Embakasi area into its ……… types of health facilities in the setting.  But we decided to retain them because …….. with the prior studies conducted in Kenya. | Citations have been included in the relevant areas |  |
| 11. | Simply clear the rationale of using the multistage purposive sampling approach, and simple Ordinary Least Squares (OLS) regression. | The sampling strategy that we used was multistage purposive sampling approach rather than purposive. We took samples in stages using smaller and smaller sampling units at each stage. We then randomly sampled the women in each stage at the selected health facilities within the sub-County.  We used LS regression because the dependent variable was a continuous variable with a normal distribution. | Page 5 |
| 12. | Give a clear idea about the Dependent Variable: The person-centered maternity score (PCMC Score). | More detail has been provided about the dependent variable the PCMC score as seen pasted below  *The PCMC scale is a validated 30-item scale with three sub-scales i) dignity and respect, ii) autonomy and communication and iii) supportive care. Each item is on a 4-point response scale – 0 “no, never”, 1: “yes, a few times”, 2: “yes, most of the time”, and 3: “yes all the time.” The full list of items are provided in (additional file 1). ……*  *Summing response to items (after reverse coding negatively worded items) yields a score range of 0 to 90, with a lower score implying poorer PCMC. To account for missing responses to certain questions (which were not applicable to certain women),- the scores were calculated using a running mean across items, and then converted to the typical summed score (0 to 90) to enable comparisons to previously published work on the scale (25),(28). We used a similar approach to generate sub-scale scores. All sub-scale scores were standardized to range from 0 to 100 to enable comparisons across sub-scales.* | Page 6 and 7 |
| 13. | How did you test the collinearity problem? Please explain. | Collinearity was tested by calculating the centered variance inflation factor (VIF) as a post-estimation. We applied the rule of thumb from Chatterjee and Hadi (2012), meaning that there was evidence of collinearity if, of all the coefficients included in the model, the largest VIF is greater than 10. In our final model, the largest VIF is 3.85. We have added a reference to the VIF in the text and the acronym list. | Page 8 paragraph 1 & Acronym list |
|  | **RESULTS SECTION** |  |  |
| 14. | Authors need to re-category the following study variables in table 1: Education Level, Education Level of Partner, Literacy, Partner's Occupation, Highest skilled person present during delivery. In these variables the sub-categories are overlapping. As for example in Education Level authors have used three sub-categories named ‘No School/Primary’, ‘Post-primary/Vocational/College’, and ‘College or above’. In this case how did the authors consider the No School and Primary in one category? Also the 2nd and 3rd options include College, so these are more puzzling. | The issue with education (of self and partner) was an error in labelling. The 2^nd^ option is defined as post-primary, vocational, and secondary. The 3^rd^ option is college or university. We thank the reviewers for identifying this; it has been corrected in Table 1. No school and primary was considered as one category due to low frequencies of response to “no school” option (only 2 respondents and 1 partner). Partner’s education responses were re-classified categories when response rates to specific options such “agricultural worker” or “unemployed” were particularly low. In the survey, the subcategories are mutually exclusive and do not overlap. For highest skilled person present at delivery, the categories are 1) Low or no skill (Auxiliary nurse, auxiliary midwife, or no skilled person); 2) Some skill (Clinical officer, nurse, midwife); 3) Very high skill (Doctor). We understand that the labels were confusing so they have been corrected to show that the subcategories are not overlapping. | Table 1 |
| 15. | What is the difference between Pregnancy Complications and severe Pregnancy Complications? You may point out some name of the Pregnancy Complications and severe Pregnancy Complications. | We have provided an explanation distinguishing between pregnancy complications and severe pregnancy complications. There were 23 pregnancy complications that the respondent could have identified, or they had the option to list a complication which was not included in the options of responses. If they listed any complications, they were asked if the problem was severe. So the severity of the problem was self-determined. This has been added in the description of the variables. | Results Section - Page 7 |
| 16. | In table 2 and 3 some variables don’t represent the exact total figure (307). Why? Make it clear. | In Table 2, some variables do not represent the exact total figure due to missing responses to individual questions, or questions which were not applicable to certain respondents (for example, if a woman had a scheduled caesarian birth, the question of choice of delivery position is not relevant to her). This is described in the Measures section, PCMC subsection (“To account for missing responses…”). We have included a note below the table which repeats this information. | Note below Table 2 |
| 17. | In table 4 authors used REML. What does it mean? Add the meaning of ‘REML’ in notes section under the table 4 and in abbreviation list. | REML means the model was fit via restricted maximum likelihood. This was already referenced in the text but the acronym has been added to the text and abbreviation list. It is not needed in Table 4 so it has been removed. | Abbreviation list |
| 18. | In the note section under the table 4 authors used ‘*** p<0.001’, this is unnecessary. | A column for p value has been added and the asterisks have been removed accordingly. | Tables |
| 19. | Authors may add an extra column in table 1 & 4 to indicate the p-values. | A column for p value has been added and the asterisks have been removed accordingly. | Table 1 and 4 |
|  | **DISCUSSION SECTION** |  |  |
| 20. | Authors may write Embakasi instead of Kenya in 2nd line of the 1st paragraph of the Discussion Section. |  |  |
| 21. | Add the reference(s) after the following sentences:  It is encouraging to note that …… that health care workers treated them with respect.  The prior studies in rural and urban Kenya ….. providers never called them by name.  Also related to the sub-optimal supportive ……. They could to control their pain. | The sentence “…It is encouraging to note that …… that health care workers treated them with respect….” Represents the results from the current study and hence does not need to be cited  The relevant citation (32) for this sentence has been included in the text  The prior studies in rural and urban Kenya ….. providers never called them by name.  A citation has been included for the phrase “…Also related to the sub-optimal supportive ……. They could to control their pain….’’ | Pg 17  Pg 18  Pg 18 |
| 22. | What do LMIC and SES mean? Clear the meaning of them and add in abbreviation list. | We have included this in the abbreviation list.  LMIC- low and middle-income countries  SES- Socioeconomic status | Abbreviation list |
|  | **FINAL COMMENTS** |  |  |
| 23. | The results indicated that women who were literate reported significantly higher levels of PCMC than women who were illiterate or semi-literate. Women whose delivery was undertaken by an unskilled birth attendant reported lower levels of PCMC than women whose delivery was conducted by a Nurse/Midwife/Clinical Officer. PCMC was also lower for women with delayed antenatal care, with those having their antenatal clinic visit in the second or third trimester, reporting lower PCMC scores than women whose first antenatal visit was in the first trimester. Finally, women who were interviewed by phone reported PCMC scores that were lower than those interviewed face-to-face at the health facility.  It is pretty obvious that literate women whose delivery was undertaken by a Nurse/Midwife/Clinical Officer and whose first antenatal visit was in the first trimester will show high PCMC scores than the others. This is a well-known fact. So, why the authors have tried to justify these findings? | We noted this to highlight that our findings are consistent with previous evidence that emphasizes the role of literacy as a factor related to high PCMC in a peri-urban setting |  |

**JOURNAL REQUIREMENTS**

|  | **COMMENTS** | **RESPONSES** | **PLACE IN MANUSCRIPT** |
| --- | --- | --- | --- |
| 1. | Please ensure that your manuscript meets PLOS ONE's style requirements, including those for file naming. | We have ensured that the manuscript meets the PLOS One style requirements. The file names have been adjusted accordingly | Throughout the manuscript |
| 2. | In the Methods section of the manuscript please include additional information on the following:  1) please provide a justification for the sample size used in your study, including any relevant power calculations (if applicable).  2) Please include in your Methods section (or in Supplementary Information files) the participating hospitals/institution. | We have included in an additional file the participating hospitals.  1)The sample was based on the available health facilities in the Embakasi area  2)The facilities were selected to represent the three categories of faith-based health facilites-Ruben Centre Clinic, public health facilities-Mama Lucy Kibaki Hospital and private health facilities-Pipeline Nursing Home, Dandora Medical Centre, Samaritan Medical Services, Mkunga Hospital, Provide International Clinic, Paradise Health Clinic. This information has been included in a supplementary information file. | Methods section |
| 3. | We note that you have indicated that data from this study are available upon request. PLOS only allows data to be available upon request if there are legal or ethical restrictions on sharing data publicly. For information on unacceptable data access restrictions, please see<http://journals.plos.org/plosone/s/data-availability#loc-unacceptable-data-access-restrictions>.    In your revised cover letter, please address the following prompts:    a) If there are ethical or legal restrictions on sharing a de-identified data set, please explain them in detail (e.g., data contain potentially identifying or sensitive patient information) and who has imposed them (e.g., an ethics committee). Please also provide contact information for a data access committee, ethics committee, or other institutional body to which data requests may be sent.    b) If there are no restrictions, please upload the minimal anonymized data set necessary to replicate your study findings as either Supporting Information files or to a stable, public repository and provide us with the relevant URLs, DOIs, or accession numbers. Please see<http://www.bmj.com/content/340/bmj.c181.long> for guidelines on how to de-identify and prepare clinical data for publication. For a list of acceptable repositories, please see<http://journals.plos.org/plosone/s/data-availability#loc-recommended-repositories>. | We have changed this and have put in a data statement that responds to the data repository where we have shared deidentified data |  |
| 4. | Your ethics statement should only appear in the Methods section of your manuscript. If your ethics statement is written in any section besides the Methods, please move it to the Methods section and delete it from any other section. Please ensure that your ethics statement is included in your manuscript, as the ethics statement entered into the online submission form will not be published alongside your manuscript. | We have included the ethics statement in the Methods section of the manuscript.  Ethics approval for the study was provided by the Strathmore University Institutional Ethics Review Committee (SU-IERC), the National Commission for Science and Technology (NACOSTI) as well as the Country Directors of health in charge of the sub-county. | Methods section |
| 5. | We noticed you have some minor occurrence of overlapping text with the following previous publication(s), which needs to be addressed:  -<https://reproductive-health-journal.biomedcentral.com/articles/10.1186/s12978-017-0449-4>  -<https://journals.plos.org/plosmedicine/article?id=10.1371%2Fjournal.pmed.1001847>  The text that needs to be addressed primarily involves the Introduction. In your revision ensure you cite all your sources (including your own works), and quote or rephrase any duplicated text outside the methods section. Further consideration is dependent on these concerns being addressed. | The overlapping text that is mentioned in the reproductive health journal are standard established frameworks of describing disrespect and abuse. We have re-framed and re-phrased the introduction with the overlapping added in text explaining why we chose to highlight the Bowser and Hill (2010) frameworks that establish disrespect and abuse. | Introduction section |
